# Supplementary material for: Patterns of Intron Gain and Loss in Fungi
Source: PLoS Biol. 2004 Nov 30;2(12):e422. doi: 10.1371/journal.pbio.0020422 (PMC532390; doi:10.1371/journal.pbio.0020422)
Supplement: Table S1 — Also available at http://genes.mit.edu/NielsenEtAl/. (4.3 MB ZIP). [file pbio.0020422.st001.zip › NielsenEtAl/html/602.html]

AN2918.1.NCU02839.1.MG01582.1.FG00558.1


```
 CLUSTAL W (1.82) Multiple Sequence Alignments - Introns Inserted


Sequence 1: MG01582.1	533 aa
Sequence 2: FG00558.1	537 aa
Sequence 3: NCU02839.1	533 aa
Sequence 4: AN2918.1	526 aa
Alignment Length: 541 aa
Number Identitical Residues: 387 aa
Alignment Score (without introns) 15849


MG01582.1 	----MATTAPAGANTANAAFR0DKEKPLAVRSSNIVAARA1VADAIRT~SLGPRGMDKM~
NCU02839.1	----MATAPAATQTASNATFR0DKEKPIAVRSSNIVAARA1VADAVRT~SLGPRGMDKM~
FG00558.1 	MSAPAMASAPAGGASSNATFR0DKEKPMAVRSSNIVAARA1VADAIRT~SLGPRGMDKM~
AN2918.1  	----MATAAPHAAPSS-TAFK0DKEKPMAVRASNILAARA1VADAIRT0SLGPRGMDKM0
          	      ::..    :: ::*: *****:***:***:**** ****:** ********** 

MG01582.1 	IRSGKGETIITNDGSTMLKSMSVMHPTAKMLVQLSNAQDVEAGDGTTSVVVICGSLLGAA
NCU02839.1	IMTGKGETIITNDGSTMLKSMSVMHPTAKMLVQLSHAQDVEAGDGTTSVVVLCGSLLGAA
FG00558.1 	IRGGKGETIITNDGNTMLKSMAVMHPTAKMLVNLSGAQDVEAGDGTTSVVVICGSLLGAA
AN2918.1  	T----------NDGNTMLRDMSVMHPAARMLVDLSAAQDVEAGDGTTSVVVIAGSLLGAA
          	           ***.***:.*:****:*:***:** ***************:.*******

MG01582.1 	DRLLSKGIHPSVISESFQRAAAASVEVLHEMSQPIALTDTAALLQAANTSLSSKIVSQYS
NCU02839.1	DRLLGKGIHPSVISEAFQRAAAAAVDVLHDMSQPISLTDTASLLQAANTSLSSKIVSQYS
FG00558.1 	DRLLSKGIHPSVISESFQRAAAAAVEVLHDMSLPITLSDTSSLLQAANTSLSSKIVSQYS
AN2918.1  	ERLLGKGIHPTVISESFQRAAAAAVEILHNMSRPINLMDRSTLLQAASTSLSSKIVSQHS
          	:***.*****:****:*******:*::**:** ** * * ::*****.**********:*

MG01582.1 	GLLGPMAVNAVTKTIDLKAADNVDLKNIRIIKRVGGTIEDSELVDGVVLTQPVLKNAGGP
NCU02839.1	NLLGPMAVNAVTKTIDLKTAENVDLKNIRIIKKVGGTIEDSELVDGLVLTQPVIKSAGGP
FG00558.1 	NLLGPMAVNSVTKTIDLKTADNVDLKNIRIVKRVGGTIEDSELVDGLVLTQPVLKNGGGP
AN2918.1  	SLLGPMAVDSVLKVVDPKTAENVDLRNIRIVKKVGGTIEDSEMVDGLVLNQGVIKSSGGP
          	.*******::* *.:* *:*:****:****:*:*********:***:**.* *:*..***

MG01582.1 	IRMEKAKIGLIQFQLSPPKPD0MENTISVNDYRQMDKIVKEERLYLLNLVKKIKKSKCNV
NCU02839.1	VRMEKARIGLIQFQLSPPKPD0MENTISVNDYRQMDKIVKEERTYLLNMVKKIKKAKCNV
FG00558.1 	ARMEKARIGLIQFQLSPPKPD0MENTIQVNDYRQMDKIVKEERLYLLNMAKKIKKAKCNV
AN2918.1  	TRIEKARIGLIQFQLSPPKPD0MENQIVVNDYRQMDKILKEERQYLLNMVKKIQKTKCNV
          	 *:***:************** *** * **********:**** ****:.***:*:****

MG01582.1 	LLIQKSILRDAVNELSLHFLAKLNILAIKDIERDEVEFICKSTGCKPIADIDSFTEDKLG
NCU02839.1	LFIQKSILRDAVNDLSLHFLQRLGILAVKDIERDEVEFICKSTGCKPIADIDSFTEDKLG
FG00558.1 	LLIQKSILRDAVNDLSLHFLAKLGILAVKDIERDEVEFICKSTGCKPIADIDSFTEDKLG
AN2918.1  	LLIQKSILRDAVNDLSLHFLSRLKILAIKDIERDEVEFLCKSLGCKPVANVDSFTEDKLG
          	*:***********:****** :* ***:**********:*** ****:*::*********

MG01582.1 	TADLIEETQSSGSRLVKITGTKS----TGKTISVVVRGANSLILDEAERSLHDALCVVRC
NCU02839.1	SADLVEEVNSSGSRMVKVTGTKS----AGKTVSVVVRGANSLILDEAERSLHDALCVIRC
FG00558.1 	YADLVEEVQSSGSRMIKVTGTKS----VGKTVSVVVRGANSLILEEAERSLHDALCVVRC
AN2918.1  	TADLVEEVQSSGARYVKITGIKAPATTANQTVSIVARGANNLILDEAERSLHDALCVIRC
          	 ***:**.:***:* :*:** *:.:::..:*:*:*.****.***:************:**

MG01582.1 	LVKKKALIAGGGAAEIEIAAQLSRRARELTGTEAICWKAFADALEVVPTTLAENAGLNSI
NCU02839.1	LVKKKALIAGGGAAEIEIASQLAKQSRALSGTEAICWKAFADAMEVIPTTLAENAGLNSI
FG00558.1 	LVKKKALIAGGGAPEIEIAAQLSKQARSLTGTEAICWKAFADALEVIPTTLAENAGLNSI
AN2918.1  	LVKKRALIAGGGAPEIEVANTLAKRARELTGTESICWKAFAEAMEVIPTTLAENAGLNSI
          	****:********.***:*  *::::* *:***:*******:*:**:*************

MG01582.1 	KVVTDLRHRHEMGEKNAGVSIKSGGVNSNIAKENVLQPLLVSTSAIELAAETVKMILRID
NCU02839.1	KVVTDLRHRHENGEKNAGVSIKSGGVNPNIAKENVLQPLLVSTSAIELAAETVKMILRID
FG00558.1 	KVVTELRHRHEMGDKNAGVSIKSGGVNTNISKENVLQPLLVSTSAIELAAETVKMILRID
AN2918.1  	KVVTDLRHRHAQGQQNAGVSIRSGGVKDDITEENILQPLLVSTSAIELAAETVKMIMRID
          	****:*****  *::******:****: :*::**:*********************:***

MG01582.1 	DIALSR
NCU02839.1	DIALSR
FG00558.1 	DIALTR
AN2918.1  	DIALSR
          	****:*
```
